# Effect of addition of FSH, LH and proteasome inhibitor MG132 to in vitro maturation medium on the developmental competence of yak (*Bos grunniens*) oocytes

**DOI:** 10.1186/1477-7827-12-30

**Published:** 2014-04-22

**Authors:** Xiao Xiao, Xiang-Dong Zi, Hui-Ran Niu, Xian-Rong Xiong, Jin-Cheng Zhong, Jian Li, Li Wang, Yong Wang

**Affiliations:** 1 College of Life Science and Technology, Southwest University for Nationalities, Chengdu 610041, People’s Republic of China; 2 College of Tibetan Plateau Research, Southwest University for Nationalities, Chengdu 610041, People’s Republic of China

**Keywords:** IVF, Yak, FSH, MG132, Early development

## Abstract

**Background:**

The competence for embryonic development after IVF is low in the yak, therefore, we investigated the effects of supplementation of FSH, LH and the proteasome inhibitor MG132 in IVM media on yak oocyte competence for development after IVF.

**Methods:**

In Experiment 1, yak cumulus-oocyte complexes (COCs) were in vitro matured (IVM) in TCM-199 with 20% fetal calf serum (FCS), 1 microg/mL estradiol-17beta, and different combinations of LH (50 or 100 IU/mL) and FSH (0, 1, 5, 10 microg/mL) at 38.6 degrees C, 5% CO_2_ in air for 24 h. Matured oocytes were exposed to frozen–thawed, heparin-capacitated yak sperm. Presumptive zygotes were cultured in SOF medium containing 6 mg/ml BSA, 0.5 mg/mL myoinositol, 3% (v/v) essential amino acids, 1% nonessential amino acids and 100 μg/mL L-glutamine (48 h, 38.5 degrees C, 5% CO_2_, 5% O_2_, and 90% N_2_). In Experiment 2, cumulus cells were collected at the end of IVM to determine FSHR and LHR mRNA expression by real-time PCR. In Experiment 3 and 4, COCs were cultured in the presence or absence of the proteasomal inhibitor MG132 from either 0–6 h or 18–24 h after initiation of maturation.

**Results:**

The optimum concentration of FSH and LH in IVM media was 5 microg/mL FSH and 50 IU/mL LH which resulted in the greatest cleavage (79.1%) and blastocyst rates (16.1%). Both FSHR and LHR mRNA were detected in yak cumulus cells after IVM. Treatment with MG132 early in maturation reduced (*P* < 0.05) cleavage and blastocyst rates. Conversely, treatment with MG132 late in maturation improved (*P* < 0.05) blastocyst rate. Optimal results with MG132 were achieved at a concentration of 10 microM.

**Conclusions:**

An optimum concentration of FSH and LH in IVM medium, and treatment with MG132 late in maturation can improve yak oocytes competence for development after IVF.

## Background

In most mammals, throughout follicular development, the oocytes undergo a series of profound changes involving both nuclear and cytoplasmic maturation induced by the pre-ovulatory surge of gonadotropins during this period [1]. These changes are essential for the formation of oocytes having the capacity for fertilization and embryonic development. Therefore, in vitro culture systems of most species often occur in in vitro maturation (IVM) media supplemented with gonadotrophins such as FSH and LH to induce cumulus cell expansion, nuclear maturation, cytoplasmic maturation, and to improve embryonic development in mice [2,3], pig [4], cow [5], equine [6] and domestic dog [7]. For gonadotrophins to act in vitro, FSH and LH receptor (FSHR and LHR) proteins and mRNA must be expressed by the cumulus cell [8]. The proteasome, a multisubunit proteolytic complex involved in degradation of ubiquitinated proteins, plays a crucial role in assuring completion of meiosis and early in maturation of oocyte, however, late in the process of oocyte maturation, the proteasome may contribute to a reduction in the functional properties of the oocyte. Treatment with the proteasome inhibitor MG132 reduced the effect of in vitro aging on oocyte competence in the mouse [9]. Furthermore, treatment of oocytes with MG132 late in maturation increased abundance of specific transcripts and improved developmental competence of parthenogenetically-activated oocytes in the pig [10] and in vitro matured oocytes in cattle [11].

The yak (*Bos grunniens*) is the principal meat and dairy animal in Qinghai-Tibet plateau, because few other animals survive in these areas [12]. In vitro production (IVP) of embryos is a well-established embryonic biotechnology with a variety of application in basic and applied sciences. IVP has been made great progress in cattle which is becoming one of the most exciting and progressive procedures available for today’s producers but the efficiency of yak IVP is still low, i.e. less than 10% of cleaved embryos (i.e., that were ≥2 cells) becoming blastocysts [13,14]. Therefore, this study was performed to investigate the effect of FSH, LH and MG132 during oocytes maturation on competence for development after fertilization in the yak.

## Methods

### Materials

Dulbecco’s phosphate-buffered saline (DPBS) was purchased from Hyclone Laboratories Inc. (Logan, UT), FSH from Bioniche Inc. (Belleville, Ontario, Canada) and fetal calf serum (FCS) from Gibco (Grand Island, NY). All other chemicals and reagents were cell-culture tested and were obtained from Sigma-Aldrich (St. Louis, MO). Synthetic oviductal fluid (SOF) medium was prepared according to the formula of Tervit et al*.*[15] minus glucose. All media were filtered through 0.2 μm Millipore filter (Carrigtwohill, Co. Cork, Ireland) and placed in an incubator (Forma Serial II, USA) to equilibrate for four to six hours in an atmosphere of 5% CO_2_ in air.

### Oocyte collection and in vitro maturation (IVM)

Collection and IVM of yak oocytes were performed in accordance with the method of Zi et al. [13,16]. Briefly, ovaries were collected from yaks at local abattoir from October to December, and transported to the laboratory in DPBS maintained at 29–33°C. Cumulus-oocyte complexes (COCs) were aspirated from follicles (2–8 mm diameter) using a hand-held 10-ml syringe connected to an 18 ga needle. COCs were collected in DPBS supplemented with 6 mg/ml BSA under a low-power (20×) stereomicroscope. Unselected COCs were rinsed three times in DPBS containing 5% (v/v) FCS and twice in TCM 199 supplemented with 20% (v/v) FCS, 1 μg/ml estradiol-17β, 100 U/ml penicillin and 100 μg/ml streptomycin, different concentrations of FSH and LH according to the experimental design (IVM medium). Only COCs having one or more layers of cumulus cells and evenly granulated ooplasm were selected. Up to 30 COCs were placed in each culture well (Nunc Inc., Naperville IL, USA) containing 600 μl of maturation medium covered with 300 μl mineral oil. COCs were allowed to mature for approximately 24 h at 38.6°C in an atmosphere of 5% CO_2_ in humidified air.

### Sperm preparation, in vitro fertilization, and embryo culture

Sperm preparation and IVF were conducted according to previously described procedures [13,16] with some modifications. Frozen semen was thawed and washed by centrifugation through a Percoll gradient (30%/45%) containing Hepes-buffered SOF medium supplemented with 5 mg/ml BSA, 50 μg/ml caffeine, 30 μg/ml glutathione and 20 μg/ml heparin (sperm medium) and centrifuged at 500 × g for 10 min to separate motile sperm. After centrifugation, 120 μl of the concentrated sperm fraction was removed and placed into 200 μl of sperm medium and incubated at 38.6°C for 15 min.

After oocyte maturation, excess cummulus cells were removed by gently swirling the COCs in a 36 mm Petri dish containing SOF with 6 mg/ml BSA (IVF medium). The COCs were further washed twice in IVF medium before being transferred into four-well plates (up to 30 per well) containing 500 μl IVF medium covered with 300 μl mineral oil per well. Each well received 50 μl of the sperm suspension (for a final concentration of 1 × 10^6^ motile sperm/ml). Oocytes and sperm were allowed to coincubate for 24–26 h at 38.6°C in an atmosphere of 5% CO_2_ in humidified air. Remnant cumulus cells were removed from the putative zygotes by gentle pipetting after approximately 26 h of coincubation. The putative zygotes were then washed three times in SOF medium. Groups of 25 embryos were cultured in 600 μl of SOF medium supplemented with 6 mg/ml BSA, 0.5 mg/ml myoinositol, 3% (v/v) essential amino acids, 1% (v/v) nonessential amino acids, 100 U/ml penicillin, 100 μg/ml streptomycin and 100 μg/ml L-glutamine (culture medium) under 300 μl mineral oil, cultured in a humidified atmosphere of 5% CO_2_, 5% O_2_, and 90% N_2_ at 38.5°C. The number of zygotes that cleaved was recorded 48 h post insemination (hpi). The culture medium was changed at 96 hpi and blastocyst development was determined on Days 7 to 9 post insemination (Day 0 = insemination). This experiment was replicated three to five times for each group.

### Expression of *FSHR* and *LHR* in cumulus cells after in vitro maturation

*GAPDH* gene was chosen as reference gene for normalizing expression levels of target genes. Primers specific for target goat genes were designed with Beacon Designer 7.0 software (Premier Biosoft International, Palo Alto, CA USA) according to manufacturers guidelines (*FSHR*: 5'-TTCAATGGGACAACGCTGATTTC-3'/5'-TGTGGCAATTAGCGTCTGAATG GA-3'; *LHR*: 5'-AGTGACACCAAGATAGCCAAGC-3/5-GGTAGAACAGGACCAGGAGG AT-3'; *GAPDH*: 5'-AGTTCCACGGCACAGTCAAG-3'/5'-ACTCAGCACCAGCATCACC- 3'). Total RNA of cumulus cells after in vitro maturation (different concentrations of FSH and LH in IVM medium) was extracted, and reverse transcribed as described previously [17]. Real-time PCR was performed using on an iCycler iQ5 Real-time Detection System (Bio-Rad, CA, USA) with the SsoFast™ EvaGreen Supermix (Bio-Rad, CA, USA) in a volume of 10 μl. The cycle parameters were 3 min at 95°C, followed by 45 cycles of denaturation at 95°C for 10 s and annealing at 60.7°C (*FSHR*), 58.9°C (*LHR*) or 57.7°C (*GAPDH*) for 10 s, and finally, melt curve analysis. The real-time PCR amplification efficiency (*E*) of each primer pair and mean Ct (threshold cycles) values were calculated and used for determination of target gene RNA transcript levels [18], which includes a correction for differences in *E* between the target and housekeeping gene. Results were expressed, however, as relative expression ratios (*RE*) rather than as fold changes from a calibrator sample. The formula, therefore, was as *RE = (1 + E ref)*^*Ct ref*^*/(1 + E target)*^*Ct target*^. Each sample was tested in triplicate, and each experiment was replicated four times with cumulus cells from 30–50 COCs for each replicate.

### Experimental design

Experiment 1 was conducted to investigate the optimal concentration of FSH and LH in IVM medium to improve yak oocyte competence for development after fertilization. COCs were matured in IVM medium that was supplemented with different combinations of LH (50 or 100 IU/ml) and FSH (0, 1, 5, 10 μg/ml).

Experiment 2 was conducted to investigate the effect of addition of FSH and LH in IVM media on *FSHR* and *LHR* mRNA expression in cumulus cells after 24 h IVM. COCs were matured in IVM medium that was supplemented with different combinations of LH (0, 50 or 100 IU/ml) and FSH (0, 1, 5, 10 μg/ml). Cumulus cells were collected at the end of IVM for each combination to determine *FSHR* and *LHR* mRNA expression.

Experiment 3 was conducted to investigate the concentration-dependent effects of MG132 added at the end of oocyte maturation on embryonic development. The MG132 was dissolved in dimethyl sulfoxide (DMSO) and was added to maturation drops so that the final concentration of DMSO was not greater than 0.5% (v/v). The control oocytes were cultured with medium supplemented with a similar amount of DMSO during IVM as for oocytes treated with MG132. COCs were matured in IVM medium (containing 5 μg/ml FSH and 50 IU/ml LH) that was supplemented with 0, 10, 20 or 30 μM MG132 from 18 h to 24 h after initiation of maturation. Treatment was achieved by washing COCs after 18 h of maturation and placing them in fresh medium containing MG132.

Experiment 4 was conducted to determine whether timing of MG132 treatment altered effects of the inhibitor on embryonic development. COCs were untreated or treated with 10 μM MG132 at two times [0–6 h of maturation (during the initiation of maturation) or 18–24 h of maturation (at the end of maturation)] using a 2 × 2 factorial arrangement of treatments. The COCs were placed in appropriate treatments at 0 h (MG132), washed at 6 h, placed in fresh IVM medium (containing 5 μg/ml FSH and 50 IU/ml LH) without MG132, washed at 18 h of maturation, and placed in fresh medium with appropriate treatment. Thus, some cultures received MG132 at 0–6 h and 18–24 h, some received MG132 from 0–6 h only, some received MG132 from 18–24 h only, and some did not receive MG132 treatment during in vitro maturation.

### Statistics

Data were analyzed statistically as follows. For each replicate, percentage of oocytes that cleaved and percentage of cleaved embryos that became blastocysts were calculated for all oocytes or embryos within the same treatment. Thus, the group of oocytes treated alike within each replicate was the experimental unit. Statistical analyses were performed using the Statistical Analysis System (version 9.2, SAS Institute Inc., Cary, NC, USA). Data were analyzed using the General Linear Models procedure. Percentage data were arcsine- transformed prior to analysis to maintain homogeneity of variance. Results are expressed as least-squares means ± standard error (SE) of the untransformed data.

## Results

### The optimal concentration of FSH and LH in IVM medium (Experiment 1)

Effects of FSH and LH concentration in IVM medium on subsequent embryonic development of the yak were shown in Table 1. The optimal concentration of FSH and LH in IVM medium was 5 μg/ml FSH and 50 IU/ml LH, i.e. both the cleavage rate and the blastocyst rate were the highest at this condition. There was a tendency for high LH concentration (100 IU/ml) in IVM medium to decrease the subsequent cleavage rate.

**Table 1:** Effects of FSH and LH concentration in IVM medium on yak embryonic development

**LH (IU/mL)**	**FSH (μg/mL)**	**No. of oocytes**	**Cleavage rate (%)**	**Blastocyst rate (%)**
50	0	233	50.6 ± 2.2^a^	10.3 ± 1.8^a^
	1	171	73.1 ± 2.9^b^	11.9 ± 1.3^a^
	5	173	79.1 ± 2.0^b^	16.1 ± 2.9^b^
	10	265	65.6 ± 2.5^c^	12.7 ± 2.8^a^
100	0	194	47.3 ± 2.5^a^	9.6 ± 1.1^a^
	1	181	58.9 ± 2.3^a^	12.3 ± 1.3^a^
	5	190	67.5 ± 1.8^c^	14.5 ± 2.6^a,b^
	10	254	64.2 ± 2.7^c^	13.4 ± 1.8^a^

Three to five replicates were performed. The blastocyst rate was calculated as the percentage of the number of cleaved embryos. Values with different letters within the same columns differ (*P* < 0.05).

### Effect of addition of FSH and LH in IVM media on FSHR and LHR mRNA expression in cumulus cells (Experiment 2)

**Figure 1:**
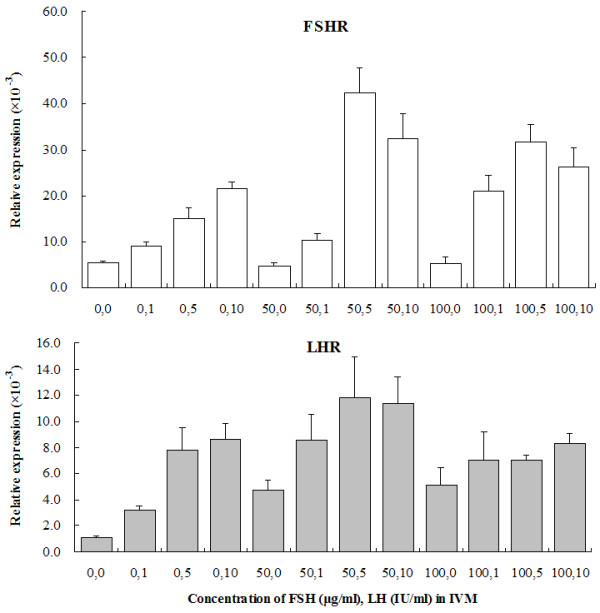
**Effect of addition of FSH and LH in IVM media on *FSHR *and *LHR *mRNA expression in yak cumulus cells after 24 h IVM as determined by real-time PCR.** The experiments were carried out in four replicates. The expression of these mRNAs was normalized to the expression of *GAPDH* measured in the same RNA preparation. Results were expressed as the mean ± SD.

*FSHR* and *LHR* mRNA expressions of cumulus cells in media supplemented with different concentrations of FSH and LH were shown in Figure 1. Both *FSHR* and *LHR* mRNA were detected in yak cumulus cells after in vitro maturation, and the greatest expression was observed when the concentration of FSH and LH in IVM medium was 5 μg/ml FSH and 50 IU/ml LH. The cumulus cells had higher numbers of *FSH* receptors than *LH* receptors. Addition of FSH in IVM media had a greater effect on increase of these receptors than that of LH.

### Concentration-dependent effect of MG132 from 18–24 h of maturation on subsequent embryonic development (Experiment 3)

Results for COCs treated from 18 to 24 h of maturation with 0, 10, 20 or 30 μM MG132 are shown in Table 2. Treatment of COCs with 10 μM MG132 in IVM medium increased (*P* < 0.05) the percentage of cleaved embryos (i.e., that were ≥2 cells) becoming blastocysts. There was, however, no effect of 10 μM MG132 on the cleavage rate. Treatment with 20 μM MG132 had no effect on the cleavage rate and the percentage of cleaved embryos becoming blastocysts. However, treatment with 30 μM MG132 had negative effect on subsequent development.

**Table 2:** Concentration-dependent effects of MG132 added from 18–24 h of maturation on yak embryonic development

**MG132 (μM)**	**No. of oocytes**	**Cleavage rate (%)**	**Blastocyst rate (%)**
0	265	76.8 ± 2.0^a^	14.1 ± 2.2^a^
5	233	77.0 ± 1.9^a^	14.7 ± 1.5^a^
10	247	79.8 ± 2.2^a^	18.1 ± 2.8^b^
20	252	72.1 ± 1.4^a,b^	12.5 ± 2.2^a,c^
30	226	69.2 ± 1.6^b^	10.6 ± 1.3^c^

Three to five replicates were performed. The blastocyst rate was calculated as the percentage of the number of cleaved embryos. Values with different letters within the same columns differ (*P* < 0.05).

### Fertilization rates of oocytes treated with MG132 from 0–6 or 18–24 h of maturation (Experiment 4)

Results are in Table 3. Addition of MG132 from 0–6 h of maturation reduced fertilization rate regardless of whether MG132 was also added at 18–24 h of maturation (*P* < 0.05), but treatment of COCs with 10 μM MG132 increased (*P* < 0.05) the percentage of cleaved embryos becoming blastocysts, as also shown in Experiment 2.

**Table 3:** Effects of timing of MG132 treatment (10 μM) on yak embryonic development

**MG132, 0–6 h**	**MG132, 18–24 h**	**No. of oocytes**	**Cleavage rate (%)**	**Blastocyst rate (%)**
Yes	Yes	184	39.1 ± 2.5^a^	11.6 ± 3.1^a^
Yes	No	178	33.0 ± 2.0^b^	9.4 ± 2.9^a^
No	Yes	172	77.9 ± 2.6^c^	19.1 ± 1.3^b^
No	No	165	75.0 ± 2.0^c^	15.0 ± 3.2^c^

Three to five replicates were performed. The blastocyst rate was calculated as the percentage of the number of cleaved embryos. Values with different letters within the same columns differ (*P* < 0.05).

## Discussion

Yak oocyte competence for fertilization and ability to support embryonic development was affected by additions of FSH, LH and the proteasomal inhibitor MG132 during the maturation process. The optimal concentration of FSH and LH in IVM media was 5 μg/ml FSH and 50 IU/ml LH. The percentage of cleaved embryos becoming blastocysts could be further improved by MG132, but actions of MG132 depended on the time of addition and the concentration. Yak oocyte competence was improved when 10 μM MG132 was added during the last 6 h maturation (from 18–24 h of maturation) and reduced when added during the first 6 h of maturation.

The mammalian oocytes are surrounded by several layers of cumulus cells, with the corona cell being closest to the oocyte. FSH and LH stimulate cumulus cell expansion, nuclear maturation, cytoplasmic maturation, and improve embryonic development in mice [2,3], pig [4], cow [5], equine [6] and dog [7]. In previous studies of mice and rats, the cumulus cells had high numbers of *FSH* receptors, but little or no *LH* receptors [19-21]. In bovines, it was reported that the FSH receptor and its mRNA were expressed in cumulus cells and granulosa cells, but not the mRNA of the *LH* receptor [22]. In this study, we observed that an optimal concentration of 5 μg/ml FSH and 50 IU/ml LH in IVM medium increased yak oocyte competence for fertilization and ability to support embryonic development (Table 1), and *FSHR* and *LHR* mRNA expressions of cumulus cells. FSH was more effective than LH due to higher *FSH* receptor levels in cumulus cells of yak oocytes than the levels of the *LH* receptor. In addition, treatment with FSH in IVM media had greater effect on the increase of *FSHR* and *LHR* mRNA expression level than that of LH (Figure 1).

Early in maturation, completion of meiosis I requires inactivation of maturation promoting factor (MPF) through a process mediated by proteasomal cleavage of ubiquitinated cyclin B1 [23]. In bovine, MG132 treatment from 0–6 h of maturation has been reported to increase the proportion of oocytes that were at metaphase I and decrease the number of oocytes that reached metaphase II at the end of maturation [11]. Therefore, inhibition of meiosis is likely a major cause for reduced oocyte competence in bovine oocytes. Inhibition of other proteasome-mediated events early in maturation may also be involved in reduced oocyte competence. For example, in the pig, MG132 can affect expression of genes involved in expansion of the extracellular matrix [24]. Similarly, in this study, we also observed that yak oocyte competence for development after fertilization was significantly reduced by addition of MG132 from 0–6 h of maturation (Table 3).

The finding that treatment of yak COCs with 10 μM MG132 late in maturation improves the percentage of cleaved embryos becoming blastocysts is consistent with other results showing beneficial effects of MG132 on aged mouse oocytes fertilized by intracytoplasmic sperm injection [9], parthenogenetically activated pig oocytes [10] and IVM bovine oocytes [11]. Beneficial effects of MG132 on nuclear remodeling, transcript abundance and embryonic development have also been shown for embryos constructed by somatic cell nuclear cloning in mammalian species [10,25-28]. Optimal results with MG132 were achieved at a concentration of 10 μM – beneficial effects were generally not observed at lower or higher concentrations (Table 2). Similar effects have been seen in mouse, goat, pig and bovine oocytes used for SCNT [25,29] and IVF [11]. In contrast to the observation by You et al. [11] in bovine oocytes, inhibition of proteasomes late in maturation can not improve the competence of yak oocyte to cleave in the present study (Tables 2 and 3). The mechanism by which MG132 late in maturation improves competence of the oocyte to support development is likely to involve arrest of processes mediated by proteasomes that ordinarily compromise the oocyte. In bovine oocytes, it was found that MG132 at the end of maturation increased relative expression of six proteins and decreased relative expression of 23 that are potentially important for oocyte competence [11].

## Conclusions

An optimal concentration of FSH and LH improves oocytes competence for development after fertilization in yak. Treatment with MG132 early in maturation reduces fertilization rate and the proportion of oocytes and cleaved embryos that became blastocysts. Conversely, inhibition of proteasomes late in maturation can improve oocytes competence for development after fertilization.

## Competing interests

The authors declare that they have no competing interests.

## Authors’ contributions

XX and XDZ carried out all aspects of the study and wrote the manuscript. HRN and XRX participated in some parts of IVF. JCZ, JL, LW and YW participated in the experimental design. All authors read and approved the final manuscript.
